# Eugenol-Induced Autophagy and Apoptosis in Breast Cancer Cells via PI3K/AKT/FOXO3a Pathway Inhibition

**DOI:** 10.3390/ijms22179243

**Published:** 2021-08-26

**Authors:** Mashan L. Abdullah, Othman Al-Shabanah, Zeinab K. Hassan, Mohamed M. Hafez

**Affiliations:** 1 Experimental Medicine Department, King Abdullah International Medical Research Center, King Saud bin Abdulaziz University for Health Sciences, MNGHA, Riyadh 11426, Saudi Arabia; 2 Pharmacology and Toxicology Department, King Saud University, Riyadh 11426, Saudi Arabia; shabanah@ksu.edu.sa; 3 Cancer Biology Department, National Cancer Institute, Cairo University, Cairo 12613, Egypt; hildahafez@hotmail.com

**Keywords:** eugenol, triple negative breast cancer, autophagy, PI3K/AKT/FOXO3a pathway

## Abstract

The use of natural compounds is promising in approaches to prevent and treat cancer. The long-term application of most currently employed chemotherapy techniques has toxic side effects. Eugenol, a phenolic phytochemical extracted from certain essential oils, has an anti-cancer effect. The modulation of autophagy can promote either the survival or apoptosis of cancer cells. Triple-negative (MDA-MB-231) and HER2 positive (SK-BR-3) breast cancer cell lines were treated with different doses of eugenol. Apoptosis was detected by a flow-cytometry technique, while autophagy was detected by acridine orange. Real-time PCR and Western blot assays were applied to investigate the effect of eugenol on the gene and protein expression levels of autophagy and apoptotic genes. Treating cells with different concentrations of eugenol significantly inhibited cell proliferation. The protein levels of AKT serine/threonine kinase 1 (AKT), forkhead box O3 (FOXO3a), cyclin dependent kinase inhibitor 1A (p21), cyclin-dependent kinase inhibitor (p27), and Caspase-3 and -9 increased significantly in Eugenol-treated cells. Eugenol also induced autophagy by upregulating the expression levels of microtubule-associated protein 1 light chain 3 (LC3) and downregulating the expression of nucleoporin 62 (NU p62). Eugenol is a promising natural anti-cancer agent against triple-negative and HER2-positive breast cancer. It appears to work by targeting the caspase pathway and by inducing autophagic cell death.

## 1. Introduction

Breast cancer has high morbidity and mortality rates among women worldwide [1]. Differences in responses to various treatments highlight the need to identify natural products that can aid cancer treatments [2]. Breast cancer is traditionally classified into several subtypes based on its biological characteristics; however, a new classification technique is based on molecular-based gene expression patterns [3] due to its heterogeneous nature [4]. Triple-negative breast cancer (TNBC) is characterized by a lack of progesterone receptor (PR), estrogen receptor (ER), and human epidermal growth factor receptor-2 (HER-2) expression [5,6]. This subtype represents approximately 15–20% of all BC with poor prognosis despite responding to conventional chemotherapy regimens [7]. Due to the lack of specific treatment guidelines, TNBC is managed with the standard treatments such as taxanes, anthracyclins, and platinum; however, such treatments leave patients associated with a high rate of relapse [8]. HER2-positive breast cancer is associated with high HER2 expression and aggressive biological behavior [4]. This subtype accounts for 15–20% of breast cancer subtypes and shows aggressive biological and clinical behavior and can metastasize to the brain and visceral organs [9]. Patients with HER2-positive tumors have a poor prognosis if not treated [10]. The available treatment options for HER2-positive breast cancer range from certain cytotoxic agents such as doxorubicin to hormonal agents [11]

Apoptosis is a type of programmed cell death, while autophagy is a self-eating process, but both processes are related. Autophagy eliminates intracellular pathogens and damaged organelles [12], thus preventing diseases [13]. As such, it can protect against prostate cancer (PC) [14,15]. It also balances energy sources during nutrient stress and starvation; it was induced among young and adult mice in response to food scarcity [16] and is stimulated by low insulin levels [17]. It is also necessary for regulating cell growth, differentiation, and function [18] as well as for preventing cell death [19]. Thus, abnormalities in this process lead to various diseases [20,21,22].

There are three types of autophagy: chaperone-mediated autophagy, micro-autophagy, and macro-autophagy. Chaperone-mediated autophagy involves soluble cytosolic protein degradation and is triggered by oxidative stress and toxic material exposure [23]. Micro-autophagy is a form of non-selective lysosomal degradation during long-lived protein turnover and is stimulated by nitrogen starvation or rapamycin [23]. Macro-autophagy regulates and transfers cellular components that are to be recycled into lysosomes via autophagosomes [24]. This mechanism of autophagy starts with the isolation of membranes that encounter extra substrates and then attach to the lysosome [25].

Apoptosis is vital to cell development and tissue homeostasis with distinct morphological changes [26], and it can be initiated by either the death receptor or the mitochondrial-dependent pathway [27]. The apoptotic pathways of cancer cells are disordered due to cell transformation, thus providing molecular targets for chemotherapeutic agents. Many natural and synthesized products exhibit anti-tumor activity by inducing the mitochondrial pathway [28].

Autophagy is a precursor of apoptosis, so inhibiting it delays apoptosis [29]. Autophagy is a non-apoptotic form of programmed cell death, and autophagy and apoptosis are often simultaneously triggered by the same stimulus. Autophagy can either inhibit or collaborate with apoptosis in tumor therapy to prevent cell death [30].

At the molecular level, several proteins are shared in apoptosis and autophagy progression. The phosphoinositide-3-kinase/AKT serine/threonine kinase 1 (PI3k/Akt) pathway plays a vital role in both apoptosis and autophagy [31]. The PI3k/Akt signal transduction pathway is frequently deregulated in many tumors and has recently emerged as a research area for drug discovery targets in oncology [32,33]. The present study evaluated the anti-cancer mechanism of eugenol, specifically considering breast cancer cell lines. This evaluation was conducted by examining autophagy leading to apoptosis. 

## 2. Results

### 2.1. The Effect of Eugenol on Cell Viability and Analysis of Population Doubling Time (PDA) 

**Figure 1:**
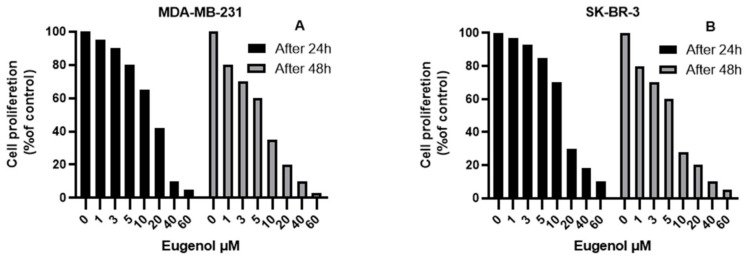
The effect of eugenol on MDA-MB-231 (**A**) and SK-BR-3 (**B**) cell viability using the MTT assay (values represent % of the control).

Figure 1 depicts the effect of eugenol on MDA-MB-231 and SK-BR-3 cell viability. The cell viability of MDA-MB-231 and SK-BR-3 was not significantly affected by eugenol concentrations up to 2.5 μM. However, at concentrations of 5, 10, and 20 μM, the viability of the MDA-MB-231 cells was significantly decreased by ~20%, 35%, and 58%, respectively, after 24 h of incubation and by ~40%, 65%, and 80%, respectively, after 48 h of incubation. High concentrations (40 and 60 μM) drastically inhibited cell viability by >90%. Meanwhile, concentrations of 5, 10, and 20 μM significantly decreased SK-BR-3 cell viability by ~15%, 30%, and 70%, respectively, after 24 h of incubation and by ~32%, 72%, and 80%, respectively, after 48 h of incubation.

**Figure 2:**
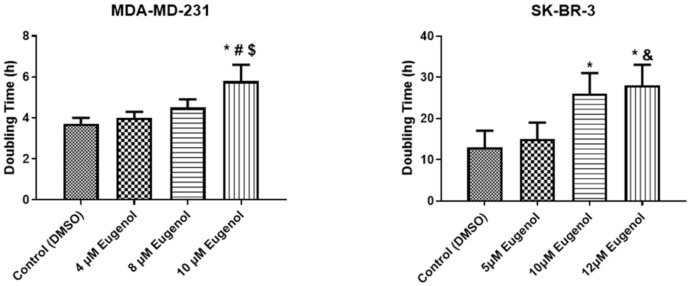
Population doubling times (PDTs) of MDA-MB-231 and SK-BR-3 cells treated with different concentrations of eugenol. The cells were seeded (2 × 10^5^ cells/well) in an E-16 plate and continuously observed for 72 h, during which time the cell index values were measured. Cell proliferation was observed at 15-min intervals. Each data point signifies an average value ± SD. All cells were analyzed in triplicate. *, #, $, and & indicate significant changes when compared to the control condition when 4 μM, 8 μM, and 5 μM of eugenol were applied, respectively (*p* < 0.05).

All of the MDA-MB-231 and SK-BR-3 cells were exposed to different concentrations of eugenol for 72 h. After counting the initial number of cells, we assessed the population doubling time (PDT), which refers to the changes in cells number and their attachment. The PDTs (mean ± SEM) of MDA-MB-231 were 4 ± 0.3, 4.5 ± 0.4, and 5.8 ± 0.8 h for eugenol concentrations of 4, 8, and 10 μM, respectively. The PDT of the same cells treated with DMSO was 3.7 ± 0.3 h. Meanwhile, the PDTs (mean ± SEM) of the SK-BR-3 cells treated with 5, 10, and 12 μM eugenol concentrations were 15 ± 4, 26 ± 5, and 28 ± 5 h, respectively. The same cells treated with DMSO had a PDT of 13 ± 3.35 h (Figure 2).

### 2.2. The Effect of Eugenol on Autophagy Induction

**Figure 3:**
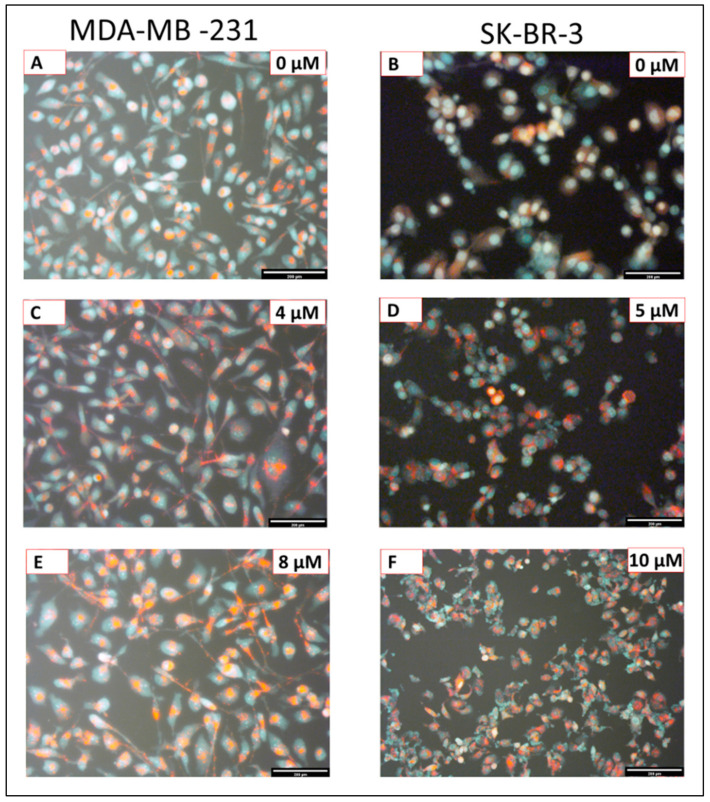
Fluorescence images of MDA-MB-231 and SK-BR-3 cells exposed to different eugenol concentrations stained with Acridine Orange (AO) under blue light excitation: untreated MDA-MB-231 (**A**) and SK-BR-3 (**B**) cells; MDA-MB-231 (**C**) and SK-BR-3 (**D**) cells treated with 4 μM and 5 μM eugenol for 24 h, respectively; MDA-MB-231 (**E**) and SK-BR-3 (**F**) cells treated with 8 μM and 10 μM eugenol for 24 h, respectively. Red fluorescence indicated autophagic vacuoles, while green fluorescence indicates the presence of nuclei. Magnification: ×10; scale bar: 200 μm.

Figure 3 illustrates how eugenol induces autophagic concentration-dependent responses. Compared to untreated cells, it generated more autophagosomes/autolysosomes and acidic vesicular organelles in both cell lines (Figure 3A,B). It also generated an increase in autophagic vesicle content in the MDA-MB-231(Figure 3A,C,E) and SK-BR-3 cells (Figure 3B,D,F) in a dose-dependent manner after 24 h of eugenol treatment.

### 2.3. The Effect of Eugenol on Microtubule-Associated Protein 1 Light Chain 3 (LC3) and Nucleoporin 62(p62) Gene Expression Levels

**Figure 4:**
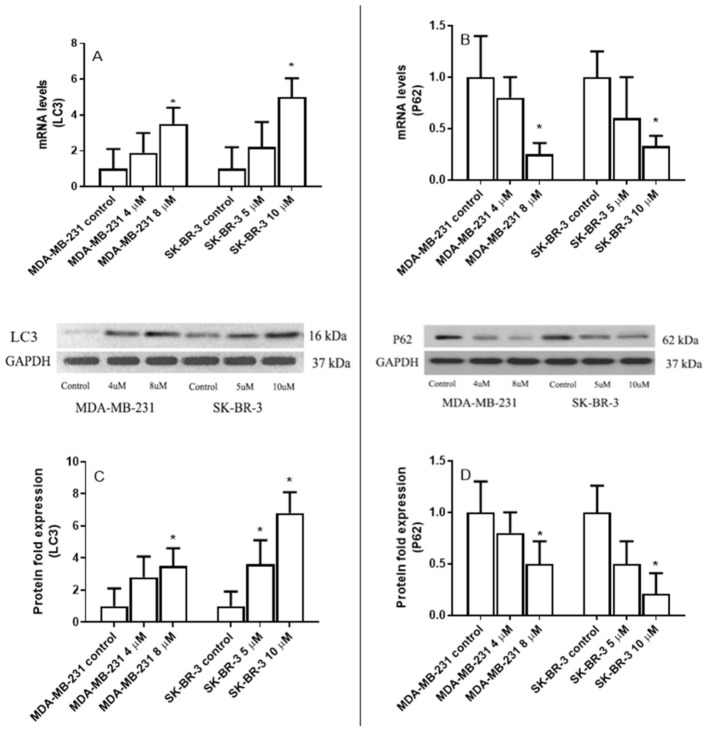
The effect of eugenol treatment on LC3 and p62 mRNA and protein expression levels. MDA-MB231 and SK-BR-3 cells were treated for 24 h (**A**,**B**). Thereafter, *LC3 and p62* mRNA expression levels were quantified by RT-PCR. The protein expression levels of LC3 and p62 were then assessed in MDA-MB231 and SK-BR3 cells treated for 24 h with 0, 4, and 8 μM and 0, 5, and 10 μM eugenol, respectively (**C**,**D**). * indicates a significant change compared to untreated cells (0 μM) at *p* < 0.05 based on a one-way ANOVA and the Tukey–Kramer post-ANOVA test.

Eugenol treatment resulted in a significant increase in LC3 mRNA levels and a significant decrease in *p62* mRNA levels, both in MDA-231 and SK-BR-3 cells (Figure 4A,B). *LC3* mRNA levels increased 1.9-fold and 3.5-fold in the MDA-MB-231 treated cells with 4 μM and 8 μM eugenol compared to untreated cells. Furthermore, the *LC3* mRNA levels of SK-B-3 increased 2.2- and 5-fold when treated with 5 μM and 10 μM eugenol, respectively. 

Conversely, *p62* mRNA levels significantly decreased in MDA-MB-231 and SK-BR-3 cells treated with eugenol. Specifically, MDA-MB-231 cells treated with 8 μM eugenol presented a 5-fold decrease in *p62* mRNA levels compared to untreated cells. Similarly, adding eugenol at a concentration of 10 μM to SK-BR-3 cells decreased *p62* expression 3.8-fold compared to untreated cells.

Similar to mRNA, MDA-MB-231 cells treated with eugenol at concentrations of 4 μM and 8 μM experienced ~2.8- and 4.5-fold increases in LC3 protein levels, respectively. Additionally, 3.6 and 6.8-fold increases in LC3 protein levels were observed in SK-BR-3 cells treated with 5 μM and 10 μM eugenol compared to untreated cells (Figure 4C).

Finally, MDA-MB-231 and SK-BR-3 cells treated with eugenol showed significant decreases in p62 expression levels. In particular, 5-fold decreases were observed in MDA-MB-231 cells treated with 8 μM eugenol and SK-BR3 cells treated with 10 μM eugenol compared to untreated cells (Figure 4D). These results indicate that both autophagy and senescence in MDA-MB-231 and SK-BR-3 breast cancer cells are collaterally induced by eugenol.

### 2.4. The Effect of Eugenol on Cyclin Dependent Kinase Inhibitor 1A (P21) Gene Expression Levels 

**Figure 5:**
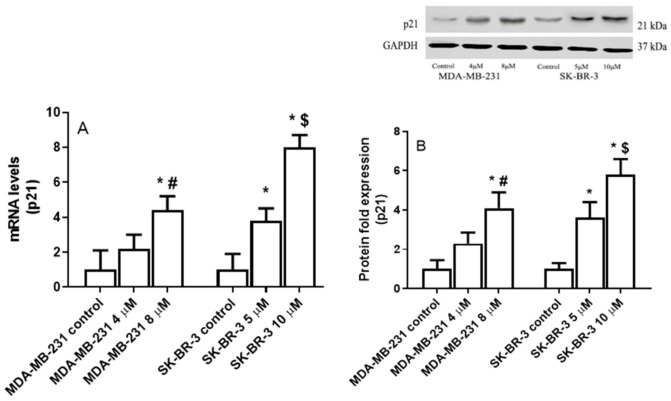
The effect of eugenol treatment on P21 mRNA and protein expression levels. (**A**) MDA-MB231 and SK-BR-3 cells were treated for 24 h. Thereafter, *P21* mRNA expression levels were quantified by RT-PCR. (**B**) MDA-MB231 and SK-BR3 cells were treated for 24 h with eugenol 0, 4, and 8 μM and 0, 5, and 10 μM eugenol, respectively. Thereafter, P21 protein expression levels were determined by Western blot analysis. * indicates significant change from untreated cells, # indicates significant difference from MDA-MB-231 at eugenol concentration 4 μM, and $ indicates significant difference from SK-BR-3 treated with 10 μM eugenol at *p* < 0.05 based on a one-way ANOVA and the Tukey–Kramer post-ANOVA test.

Figure 5A shows that eugenol treatment significantly increased *P21* mRNA levels, both in the MDA-231 and SK-BR-3 cells. Specifically, in the MDA-MB-231 cells treated with 4 and 8 μM eugenol, *P21* mRNA levels increased 2.2- and 4.4-fold compared to untreated cells. Meanwhile, no significant differences were observed when the two different concentrations of eugenol were compared. Similarly, eugenol concentrations of 5 and 10 μM induced 3.8- and 8.0-fold increases in *P21* mRNA levels in the SK-BR-3 cells, respectively.

In the MDA-MB-231 cells treated with 4 and 8 μM eugenol, P21 protein levels were upregulated by ~2.3- and 4.1-fold, respectively. Moreover, 3.6- and 5.8-fold increases in P21 protein levels were observed in SK-BR3 cells treated with 5 and 10 μM eugenol, respectively, compared to untreated cells (Figure 5B). These results suggest that eugenol induces autophagy in MDA-MB-231 and SK-BR-3 breast cancer cells.

### 2.5. The Effect of Eugenol on Forkhead box O3 (FOXO3a) Transcription Factor Gene Expression Levels 

**Figure 6:**
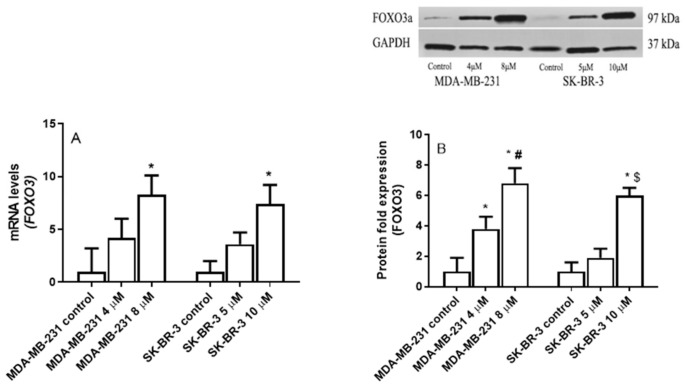
The effect of eugenol treatment on *FOXO3* mRNA and protein levels. (**A**) MDA-MB231 and SK-BR-3 cells were treated for 24 h with eugenol; thereafter, *FOXO3a* mRNA expression levels were quantified by RT-PCR. (**B**) MDA-MB231 and SK-BR3 cells were treated for 24 h with 0, 4, and 8 μM and 0, 5, and 10 μM eugenol, respectively. Thereafter, FOXO3a protein expression levels were determined by Western blot analysis. One of the three representative experiments is shown. * indicates significant difference from untreated cells, # indicates significant difference from MDA-MB-231 at eugenol concentration 4 μM, and $ indicates significant difference from SK-BR-3 treated with 10 μM eugenol at *p* < 0.05 using ANOVA followed by Tukey–Kramer post-ANOVA test.

MDA-MB231 and SK-BR-3 cells were incubated for 24 h with increasing concentrations of eugenol (0, 4, and 8 μM) to determine eugenol’s effect on FOXO3a gene expression. Thereafter, the FOXO3a mRNA and protein levels were detected using the qRT-PCR and Western blot techniques. Figure 6 clearly shows that eugenol induced *FOXO3a* in both MDA-231 and SK-BR-3. In the MDA-231 cells, 4- and 8-μM concentrations of eugenol induced 4.2- and 8.3-fold increases in *FOXO3a* mRNA levels, respectively, compared to untreated cells; no significant differences were observed between these two different concentrations (Figure 6A). At the protein level, 4- and 8-μM concentrations significantly upregulated FOXO3a protein levels by ~3.8- and 6.8-fold, respectively, compared to the untreated cells (Figure 6B). 

Similarly, eugenol concentrations of 5 and 10 μM increased *FOXO3a* mRNA levels in SK-BR-3 cells 3.6- and 7.4-fold, respectively. At the protein level, 5- and 10-μM concentrations of eugenol increased FOXO3a protein levels by ~1.9- and 6-fold, respectively.

### 2.6. The Effect of Eugenol on Cyclin-Dependent Kinase Inhibitor (p27), and AKT Serine/Threonine Kinase 1 (AKT) Expression Levels in MDA-MB-231 and SK-BR-3 Cell Lines

MDA-MB231 and SK-BR-3 cells were treated for 24 h with increasing concentrations of eugenol (0, 4, and 8 μM) to determine eugenol’s effect on p27 gene expression. Thereafter, p27 mRNA and protein levels were detected using qRT-PCR and Western blot techniques, respectively.

**Figure 7:**
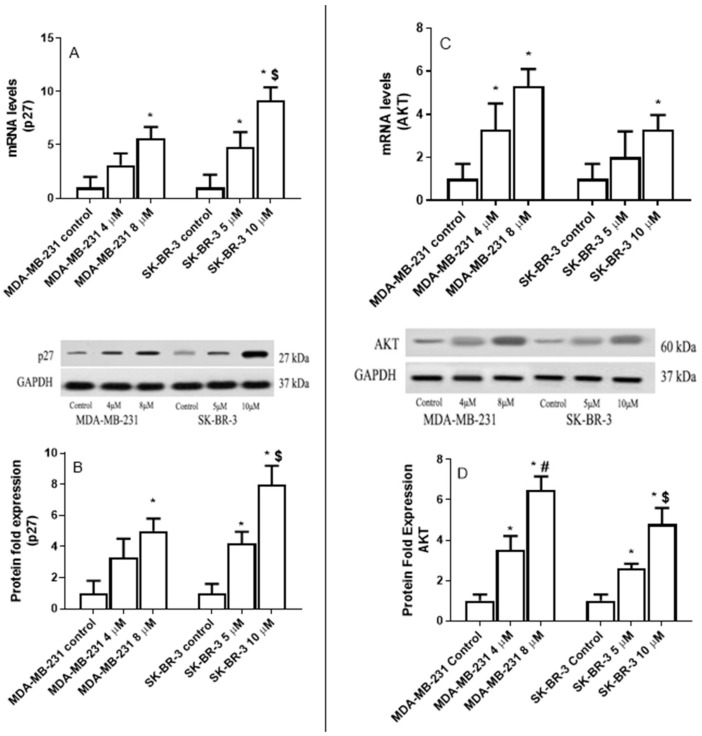
The effect of eugenol treatment on p27 and AKT mRNA and protein expression levels. MDA-MB231 and SK-BR-3 cells were treated for 24 h (**A**,**B**). Thereafter, *p27* and *AKT* mRNA expression levels were quantified by RT-PCR. The protein expression levels of p27 and AKT were then assessed in MDA-MB231 and SK-BR3 cells treated for 24 h with eugenol 0, 4, and 8 μM and 0, 5, and 10 μM eugenol, respectively (**C**,**D**). * indicates a significant change compared to untreated cells (0 μM), # indicates a significant change from MDA-MB-231 at eugenol concentration 4 μM, and $ indicates significant change from SK-BR-3 treated with 10 μM eugenol at *p* < 0.05 based on a one-way ANOVA and the Tukey–Kramer post-ANOVA test.

Figure 7 indicates that eugenol significantly increased *p27* mRNA levels in both MDA-231 and SK-BR-3. In MDA-231 cells, 4- and 8-μM concentrations induced 3.1- and 5.6-fold increases in *p27* mRNA levels compared to untreated cells (Figure 7A). Moreover, 4- and 8-μM concentrations of eugenol increased p27 protein levels by ~2.8- and 4.6-fold, respectively, compared to the untreated cells (Figure 7B).

Similarly, in SK-BR-3 cells, 5- and 10-μM eugenol concentrations increased mRNA *p27* levels 4.8- and 9.2-fold, respectively. Additionally, the 10-μM eugenol concentration increased *p27* mRNA levels significantly more than the 5-μM concentration. Furthermore, the 10-μM eugenol concentration increased the *p27* mRNA levels to a greater extent than the 4- and 8-μM concentrations did in the MDA-MB-231 cells. Similarly, the 5- and 10-μM concentrations of eugenol increased the p27 protein levels by ~2.8- and 3.8-fold, respectively. 

MDA-MB231 and SK-BR-3 cells were treated for 24 h with increasing concentrations of eugenol (0, 4, and 8 μM) to determine whether eugenol affects AKT gene expression. AKT mRNA and protein levels were verified using qRT-PCR and Western blot techniques. Figure 7C,D clearly show that eugenol induced AKT in both the MDA-231 and SK-BR-3 cells. Specifically, in MDA-231 cells, 4- and 8-μM eugenol concentrations induced 3.3- and 5.3-fold increases in mRNA expression in *AKT* compared with untreated cells. Additionally, adding eugenol to SK-BR-3 cells at a concentration of 10 μM increased the *AKT* mRNA expression levels 3.3-fold compared to untreated cells. 

Moreover, at the protein level, 4- and 8-μM eugenol concentrations caused 3.5- and 6.4-fold increases in AKT protein levels, respectively, in MDA-231 cells. Likewise, 5- and 10-μM eugenol concentrations led to 2.6- and 4.8-fold increases in AKT protein levels, respectively. 

### 2.7. The Effect of Eugenol on Caspase-3/7 and Cell Death in MDA-MB-231 and SK-BR-3 Cell Lines

In the next phase of this study, we investigated whether eugenol-induced apoptotic genes and cell death were due to increases in apoptotic and/or necrotic cells. To do this, we calculated the percentage of MDA-MB231 and SK-BR-3 cells that underwent apoptosis/death in response to increasing concentrations of eugenol using a caspase-3/7 assay and flow cytometry after 24 h of incubation with different doses of eugenol. 

**Figure 8:**
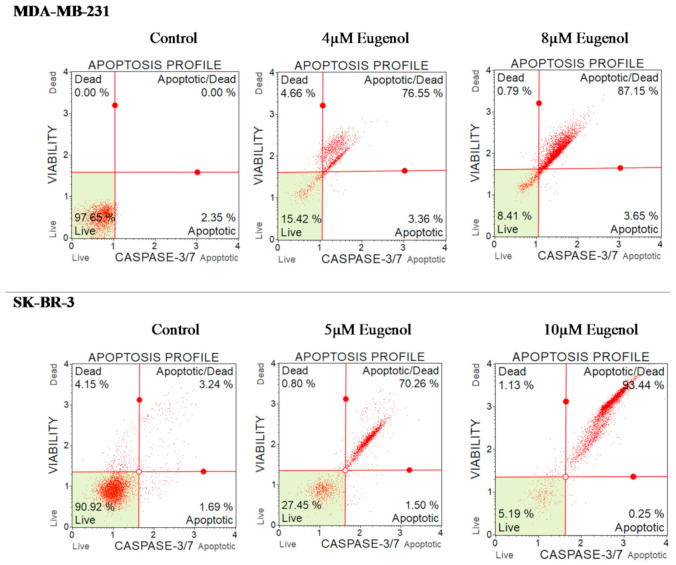
Effect of eugenol treatment on the percentage of apoptosis and caspase-3/7 in MDA-MB-231 and SK-BR-3 cells. Cells were treated for 24 h with eugenol (4 and 8 μM and 5 and 10 μM, respectively). The percentage of cells undergoing apoptosis was determined using a caspase-3/7 assay. Cells were immediately analyzed on the Muse™ Cell Analyzer (Merck KGaA Co., Darmstadt, Germany).

Figure 8 illustrates that 97.65% of untreated MDA-MB-231 cells were healthy, as were 90.2% of SK-BR-3 cells. However, the percentage of cells in late apoptosis significantly increased among treated MDA-MB-231 cells in response to eugenol. Specifically, 75.55% and 87.15% of the MDA-MB-231 cells were in late apoptosis after treatment with eugenol at concentrations of 4 and 8 μM, respectively. A similar finding was observed for SK-BR-3 cells. Only 3.24% of the untreated cells experienced late apoptosis, whereas 70.25% of the cells treated with 5 μM eugenol and 93.44% of cells treated with 10 μM eugenol experienced late apoptosis.

### 2.8. The Effect of Eugenol on the Apoptotic Gene Expression Levels

We incubated MDA-MB-231 and SK-BR-3 cells for 24 h with increasing concentrations of eugenol (4–8 μM and 5–10 μM, respectively) to investigate how these cells’ caspase-3 and -9 expression levels responded to eugenol treatment. 

*Caspase-3* and -*9* mRNA levels significantly increased in the treated MDA-MB-231 and SK-BR-3 cells depending on the concentration of eugenol that was applied (Figure 8). The maximum mRNA induction levels of *caspase-3* (6.5-fold increase) and *caspase-9* (5.8-fold) in the MDA-MB-231 cells were associated with the 8-μM eugenol concentration. Similarly, the 10-μM concentration of eugenol was associated with increases in the mRNA levels of *caspase-3* (7-fold) and *caspase-9* (6.7-fold) in SK-BR-3 cells. 

**Figure 9:**
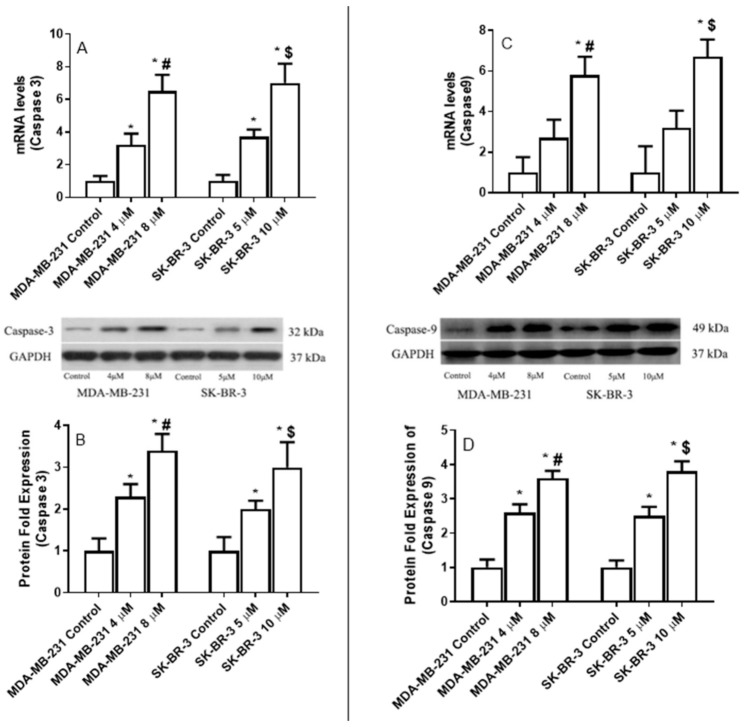
The effect of eugenol treatment on caspase-3 and caspase-9 mRNA and protein expression levels. MDA-MB231 and SK-BR-3 cells were treated for 24 h (**A**,**B**). Thereafter, *caspase-3* and *caspase-9* mRNA expression levels were quantified by RT-PCR. The protein expression levels of caspase-3 and caspase-9 were then assessed in MDA-MB231 and SK-BR3 cells treated for 24 h with 0, 4, and 8 μM and 0, 5, and 10 μM eugenol, respectively (**C**,**D**). * indicates a significant change compared to untreated cells (0 μM), # indicates a significant change from MDA-MB-231 at eugenol concentration 4 μM, and $ indicates significant changes from SK-BR-3 treated with 10 μM eugenol at *p* < 0.05 based on a one-way ANOVA and the Tukey–Kramer post-ANOVA test.

Eugenol (8 μM) also induced 3.4-fold and 3.6-fold increases in the protein expression levels of caspase-3 and caspase-9, respectively, in SK-BR-3 cells. At a concentration of 10 μM, eugenol induced 3- and 3.8-fold increases in caspase-3 and caspase-9 expression levels, respectively, in SK-BR-3 cells. These findings confirm that eugenol induces apoptosis, both in triple-negative and HER2-positive breast cancer cells (Figure 9).

## 3. Discussion

Eugenol is a phenylpropanoid compound used as a local anesthetic and antiseptic in medicine and as a flavoring agent in food products. However, it has toxic effects when consumed in high concentrations. The present study aimed to investigate the extent to which eugenol induces autophagy and apoptosis against the MDA-MB-231 and SK-BR-3 breast cancer cell lines. 

The results of the present study indicate that eugenol had concentration- and time-dependent proliferation effects on breast cancer cell lines, suggesting its potential anti-proliferative and apoptotic effects on MDA-MB-231 and SK-BR-3 cells. Proliferation was determined via MTT assay and indicated that eugenol concentrations at 40 and 60 μM inhibited cell proliferation by more than 90% in both MDA-MB-231 and SK-BR-3.

In line with this finding, the data related to population doubling time (PDT) also demonstrate that higher eugenol concentrations led to increased cell population doubling times compared to control cells, which, in fact, require a shorter population doubling time. These data clearly represent the direct inhibitory effect of eugenol on both cell lines under investigation. Similarly, Al-Sharif et al. found that 4 μM eugenol could inhibit cell proliferation in ER-negative and ER-positive breast cancer cells [34]. Data from other breast cancer cell lines such as MDA-MB231 show that cell migration and proliferation are affected by time and eugenol concentration. Eugenol treatment shows an anti-metastatic effect by reducing the invasion and migration of MDA-MB-231 breast cancer cells mostly by reducing the expression of MMP-2 and MMP-9 [35]. The literature presents variations in the severity of the cytotoxic effect of eugenol, which could result from variations in the eugenol concentrations used, purity, or types of cell lines used. 

Autophagy is an essential metabolic process that controls the energy balance between individual cells as well as an entire organism, and it represents any given cell’s response to stress [36]. It also plays an essential role in intracellular quality control and homeostasis under normal conditions and plays a dual role in cytoprotection and cell death during stress [37].

The data presented in this study indicate that eugenol performs anti-cancer activity by inducing autophagy, as evidenced by the accumulation of MDC and AO in eugenol-treated MDA-MB231 and SK-BR-3 cells. The expression of several autophagy-associated proteins was evaluated, and it was found that LC3 expression was markedly induced by eugenol in treated MDA-MB231 and SK-BR-3 cells. Additionally, eugenol exhibited great potential to reduce autophagy inhibitor expression, clearly indicating its role in triggering autophagy. 

p62 is a ubiquitin and LC3 binding protein, and it interacts with LC3 and is degraded alongside poly-ubiquitinated proteins destined for autophagosomes. Therefore, p62 protein levels decrease when autophagy is induced [37]. Several studies have demonstrated the critical role that p62 can play in different cellular functions and cancer. Moreover, p62 can act as a cellular signaling switch through recruiting and oligomerization with cellular molecules to control cell survival, apoptosis, and autophagy [38,39]. Mathew et al. (2009) confirmed that the elimination of p62 by autophagy can suppress tumorigenesis and that compromised autophagy leads to p62 accumulation [40]. Similarly, data from autophagy-deficient mice suggests a link between autophagy and p62 [41]. These data suggested that p62 may play an essential role in cell apoptosis or autophagy. The expression levels of P62 were investigated to elucidate the relation between eugenol-induced autophagy and apoptosis in breast cancer cells. In this study, p62 expression was decreased in MDA-MB231 and SK-BR-3 cells treated with different concentrations of eugenol. In addition, eugenol increases P21 mRNA and protein expression levels. Another study reported that p62 depletion can inhibit the recruitment of LC3 to autophagosomes under starvation conditions and that the background level of LC3II is exceptionally high in cells that overexpress p62. Such findings suggest that high p62 levels intensify autophagic activity [42]. 

The P21 gene is directly regulated by the P53 gene and is involved in the P53-mediated DNA-damaging response. Several studies have demonstrated that P21 has anti-tumor activity. Additionally, it is known as cyclin-dependent kinases (CDK) inhibitor and interferes with cell cycle progression by inhibiting the formation of the cyclin–CDK complex [43]. In prostate cancer cells, it has been shown that the upregulation of P21 using a natural compound such as oridonin can induce cell autophagy [44]. Therefore, we inspected the mRNA level and protein expression of P21 after treating breast cancer cells with eugenol. We found that P21 expression was promoted by eugenol, in a dose-dependent manner in MDA-MB-231 and SK-BR-3 cell lines. These results indicate that autophagy may be induced by eugenol in MDA-MB-231 and SK-BR-3 breast cancer cells. These results align with previous findings suggesting that autophagy-associated protein levels increase in cells treated with nanomaterials [45]. Similarly, Fujiwara K et al. demonstrated that P21 can ascertain the type of cell death, either apoptosis or autophagy [46]. Eugenol is a cytotoxic compound that can trigger cell death in cancer cells via the caspase pathway. In the current study, late apoptosis was induced in breast cancer cell lines by eugenol in a dose-dependent manner. As in our previous study, caspase-3, -7, and -9 expression levels were overexpressed in both breast cancer cell lines along with increasing apoptosis [35].

A previous study suggested that eugenol suppresses E2F1/survivin and triggers apoptosis in breast cancer cells [34]. Thus, eugenol can enhance Bax, thereby increasing cytochrome C while activating the caspase pathway required for apoptosis [47]. Another study showed that eugenol might cause DNA degradation or cracking in breast cancer cell lines and can provoke a robust cytotoxic response [48]. Similarly, eugenol induces apoptosis in human osteosarcoma cells by activating caspase-3 [47]. Meanwhile, cells deficient in caspase-3 or -7 exhibited delayed mitochondrial events related to intrinsic apoptosis, thus suggesting anti-tumor activity [49]. 

Forkhead box O1 (FOXO1) and FOXO3 are FOXO transcriptional protein family members involved in autophagy modulation, and they are connected in autophagy induction. FOXO3 transcriptionally activates the class I PI3K catalytic subunit PIK3CA, which, in turn, phosphorylates and upregulates AKT1 activity. Then, activated AKT1 catalyzed FOXO1 phosphorylation results in FOXO1 translocation to the cytoplasm, which leads to autophagy [50]. It is now well established from a variety of studies that alterations in the Akt/FOXO3a pathway regulates autophagic cell death and may result in the development of cancer [51,52,53]. Inactivation or the knocking out of autophagy-related genes leads to tumorigenesis in mammal models, whereas the overexpression of autophagy-related genes suppresses the tumorigenesis in breast, ovarian, and prostate cancer [51,54]. It is known that the Akt/FOXO3a pathway can modulate autophagy in skeletal muscles by activating LC3 [55]. However, it is unclear whether eugenol exerts effects in breast cancer cells by modulating the Akt/FOXO3a pathway and regulating autophagy.

In the present study, eugenol treatment increased AKT expression in MDA-MB-231 and SK-BR-3 cells in a dose-dependent manner. Previous research indicates that FOXO3 can transcriptionally activate autophagy by upregulating autophagy-related (ATG) genes or autophagy regulatory genes [56,57]. However, other previous research has also found that FOXO3 induces FOXO1-dependent autophagy by activating the AKT1 signaling pathway [50]. 

With regard to the research methods, some limitations need to be acknowledged; we only tested two types of breast cancer cells; however, these results may not apply to all types of cancer cells. Additionally, the results require more mechanistic study by using a specific autophagy activator or inhibitor to further approve the ability of eugenol in inducing autophagy. Unfortunately, the current study was unable to investigate cleaved caspase-3 and cleaved caspase-9 due to antibody issues.

## 4. Materials and Methods

### 4.1. Cell Viability Assay Using MTT

The viability of breast cancer cells (triple negative-MDA-MB-231 (ATCC HTB-26^®^) and HER2 positive-SK-BR-3 (ATCC HTB-30™) (Rockville, MD, USA)) in response to different concentrations of eugenol (Sigma Aldrich, St. Louis, MO, USA) was determined via 3-(4,5-dimethylthiazol-2-yl)-2,5-diphenyl tetrazolium bromide (MTT) assay (Roche Diagnostics, Mannheim, Germany).

Triple-negative and HER2positive cell lines were seeded in 96-well plates (1 × 104 cells/well) and were incubated for 24 h. Each well was incubated in a fresh medium containing different concentrations of eugenol; untreated cells were treated with dimethyl sulfoxide (DMSO). After 24 h, breast cancer cell proliferation was detected by adding 10 μL of MTT (5 mg/mL in PBS) reagent to each well, and the plates were incubated for 4 h at 37 °C. Then, 100 μL of isopropanol was added to each well, and after 15 min. the amount of formazan was quantified by measuring absorbance at 450 nm using an ELISA reader. The percentage of cell proliferation was calculated relative to the untreated wells designated as 100% viable cells, where % cell proliferation = (A treated)/(A untreated cells) × 100. All of the experiments were repeated three times. 

### 4.2. Cell Doubling Time Analysis Using a Real-Time Cell Electronic Sensing System

The next step was to measure the effects of different concentrations of eugenol on the time required for MDA-MB-231 and SK-BR-3 cell doubling. To do this, we used 100 μL of a complete medium containing 2 × 104 cells in each well of microtiter plates and inserted them into a real-time cell electronic sensing system (ACEA Biosciences Inc., San Diego, CA, USA) after 30 min of incubation in a CO_2_ incubator. When the cells reached the log phase, we added different concentrations of eugenol to each well and then incubated the samples under electronic monitoring for 72 h.

### 4.3. Autophagy Rate Determination by Acridine Orange (AO)

AO is a compound used for a staining method to determine autophagy rates. Briefly, cells were seeded in six-well plates at 1 × 106 cells/mL and were incubated for 24 h. After eugenol treatment, AO (Life Technologies, Grand Island, NY, USA) was added to reach a final concentration of 0.5 μg/mL, and the mixture was incubated in a dark field for 10 min. Then, the stained cells were washed twice with PBS, resuspended in 200 μL of a buffer, and analyzed. Following the staining procedure, red fluorescence indicated autophagic vacuoles, while green fluorescence indicated the presence of nuclei. Additionally, the AO-stained cells were plated, and the autophagic vacuoles were observed using fluorescence microscopy (Olympus Southall, UK). 

### 4.4. RNA Extraction and Quantitative Reverse Transcriptase-Polymerase Chain Reaction (qRT-PCR)

MDA-MB-231 cells were treated with 4 and 8 μM eugenol, whereas SK-BR-3 cells were treated with 5 and 10 μM eugenol for 24 h. These doses and time were chosen based on the cytotoxicity results. We conducted qRT-PCR to determine the effect of eugenol on gene expression levels. Total RNA extraction was conducted using TRIzol reagent (Invitrogen^®^, Waltham, MA, USA) according to the standard protocol [58]. The quality and quantity of the isolated RNA were assessed by measuring the absorbance at 260 nm and by maintaining a 260/280 ratio of ~2.0. The cDNA was synthesized from 1 μg total RNA using a high-capacity cDNA reverse transcription kit (Applied Biosystems^®^) per the manufacturer’s instructions. 

We performed real-time PCR using gene expression master mix (Applied Biosystems, Foster City, CA, USA) on the ABI PRISM 7500 Detection System (Applied Biosystems, USA) to detect the expression levels of FOXO3 (Hs00818121_m1), P21 (Hs01040810_m1), cyclin-dependent kinase inhibitor 1B (P27Kip1), AKT (Hs00178289_m1), caspase-3 (Hs00234387_m1), caspase-9 (Hs00962278_m1), MAP1LC3A (Hs01076567_g1), nucleoporin 62 (Hs02621445_s1), and BCL2 interacting protein 3 (Hs00969291_m1). Glyceraldehyde-3-phosphate dehydrogenase (Hs02786624_g1) was used as an internal control. Each sample was analyzed in triplicate, and representative data sets are shown. Results were analyzed using the 2^−ΔΔCT^ method. Data were expressed as mean fold changes ± standard deviation (SD) for three independent amplifications.

### 4.5. Western Blot Analysis

All of the protein was extracted from the cells, and its concentration was determined using NanoDrop (NanoDrop 8000, Thermo Scientific, Wilmington, DE, USA). Western blot analysis was performed using a previously described method [35]. Briefly, 30–50 μg of protein from the treated and untreated cells were separated by 10% sodium dodecyl sulfate (SDS)-polyacrylamide gel electrophoresis (PAGE) before being electrophoretically transferred to a PVDF membrane. Protein blots were then blocked overnight at 4 °C in a blocking solution and were subsequently washed several times with TBS-Tween-20.

Following this, the samples were incubated in a TBS solution with a primary antibody against target proteins. They were then further incubated for 2 h at room temperature with peroxidase-conjugated IgG secondary antibodies for 2 h at room temperature. The bands were digitally visualized using the chemiluminescent substrate, and images were captured using Quantity One software (Bio-Rad, Hercules, CA, USA). The total protein images of the treated cells were compared with those of the untreated cells after normalization to GAPDH. We conducted a relative quantification of the protein levels by selecting bands with triplicate runs, and the mean value with SD was reported. 

### 4.6. Flow Cytometric Analysis of Apoptosis and Caspase-3/7 Activity

A caspase-3/7 Kit (Muse caspase-3/7 kit Millipore, Burlington, MA, USA) was used to measure caspase-3 and caspase-7 activation according to the manufacturer’s instructions as a means to detect early and late apoptosis. In brief, MDA-MB-231 and SK-BR-3 cells were treated with different doses of eugenol for 24 h. The medium was then removed, and the cells were washed with cold PBS. After trypsinization, the cells were collected by centrifugation at 300× *g* for 5 min and were then resuspended in 0.5 mL PBS. After the working solution was added to the cell suspension, the suspension was incubated at 37 °C for 30 min. Then, 150 µL of the 7-ADD working solution was added to the cell suspension, which was subsequently incubated in the dark for 5 min. Afterward, the suspension was analyzed using the Muse™ Cell Analyzer (Merck KGaA Co., Darmstadt, Germany). The viable, apoptotic, and dead cell populations were determined.

### 4.7. Statistical Analyses

Comparative analysis was performed using GraphPad 5.0 Prism software (GraphPad Software, Inc., La Jolla, CA, USA). Data were expressed as means ± SD of N observations’ means. All of the experiments were repeated three times, and all of the data were compiled from a minimum of triplicate experiments. One-way analysis of variance (ANOVA) followed by the Tukey–Kramer post-ANOVA test were used to assess all of the treated groups that were significantly different from the untreated cells. The significance level was set at *p* < 0.05.

## 5. Conclusions

In conclusion, the current data indicate that eugenol is a promising natural anti-cancer agent against triple-negative and HER2-positive breast cancer. It appears to work by targeting the caspase pathway and inducing autophagic cell death and by modulating the expression of several key proteins. Thus, this study underlines the potential utility of eugenol-induced cell death as a new cancer treatment modality. Nevertheless, the precise mechanism of eugenol against cancer cells remains to be elucidated. 

Consequently, this research has introduced many questions in need of further investigation such as examining the long-term therapeutic efficacy and safety of eugenol; further research could also be conducted to determine the effectiveness of eugenol on autophagic process and apoptosis in the presence of commonly used anticancer treatments. In addition, more studies are needed to better understand the complex linkages between autophagy and apoptosis and to investigate more proteins involved in autophagy such as beclin1, ATG, and LC3I/LC3II conversion. 

## Data Availability

All of the data used in this study have been provided in the main text.
